# Controlling viscous fingering instabilities of complex fluids

**DOI:** 10.1038/s41598-024-52218-w

**Published:** 2024-01-29

**Authors:** Alban Pouplard, Peichun Amy Tsai

**Affiliations:** Department of Mechanical Engineering, University of Alberta, Edmonton, AB T6G 2G8 Canada

**Keywords:** Engineering, Fluid dynamics

## Abstract

Despite their aesthetic elegance, wavy or fingering patterns emerge when a fluid of low viscosity pushes another immiscible fluid of high viscosity in a porous medium, producing an incomplete sweep and hampering several crucial technologies. Some examples include chromatography, printing, coating flows, oil-well cementing, as well as large-scale technologies of groundwater and enhanced oil recovery. Controlling such fingering instabilities is notoriously challenging and unresolved for complex fluids of varying viscosity because the fluids’ mobility contrast is often predetermined and yet the predominant drive in determining a stable, flat or unstable, wavy interface. Here we show, experimentally and theoretically, how to suppress or control the primary viscous fingering patterns of a common type of complex fluids (of shear-thinning with a low yield stress) using a radially tapered cell of linearly varying gap thickness, *h*(*r*). Experimentally, we displace a complex viscous (PAA) solution with gas under a constant flow rate (*Q*), varied between 0.02 and 2 slpm (standard liter per minute), in a radially converging cell with a constant gap-thickness gradient, $\alpha = dh/dr < 0$. A stable, uniform interface emerges at low *Q* and in a steeper cell (i.e., greater $|\alpha |$) for the complex fluids, whereas unstable fingering pattern at high *Q* and smaller $|\alpha |$. Our theoretical predictions with a simplified linear stability analysis show an agreeable stability criterion with experimental data, quantitatively offering strategies to control complex fluid-fluid patterns and displacements in microfluidics and porous media.

## Introduction

The process of one fluid pushing another is ubiquitous while involving fascinating and complex patterns stemming from flow instabilities. During immiscible displacement of more-viscous fluid pushed by another less-viscous one in a porous media, the mobility contrast results in the tunneling of the displacing fluid and hence fingering patterns, hindering a full swipe of the displaced fluid. As the utmost detrimental, limiting factor in enhanced oil recovery processes, this so-called viscous fingering or Saffman–Taylor instability^1–5^ has been extensively studied since the 1980s. In particular, a convenient paradigm of Hele−Shaw cells consisting of two parallel plates spaced with a constant gap thickness yielding a homogeneous permeability is often used to understand the effects of inertia, gravity, and rotation. Recent studies using simple fluids of constant viscosity have considered centrifugally driven viscous fingering via rotation^6^ and found that the inertia tends to increase the finger-width^7^ and curvature-dependent surface tension can theoretically lead to the stabilization (destabilization) of conventionally unstable (stable) situations^8^. In the last two decades, studies of viscous fingering have been extended to complex fluids, usually leading to wider fingers compared to the simple Newtonian counterparts^9^. Intriguing side-branching patterns with multiple small sided-fingers are often observed with complex, yield-stress fluids^10^ in a uniform cell.

The control of the fingering instabilities plays a significant role in enhancing the efficiency of various technological applications, e.g., chromatography separation^11^, printing devices^12^, coating^13^, oil-well cementing, and large-scale enhanced oil recovery^14^. For simple Newtonian fluids, several strategies have recently been developed to suppress the fingering instability, for example, using time-dependant injection flow rate^15–17^, an elastic confinement^18–20^, a gap-gradient cell^21–23^, and an external electric field^24^. Nevertheless, such crucial control of the primary viscous fingering instability has not been reported for complex fluids, which are commonly present in natural and industrial settings. Here, we demonstrate the feasibility of inhibiting the viscous fingering instability of complex fluids of shear-thinning with a low-yield stress using a radially-tapered narrow cell using experiment and theory.

**Figure 1:**
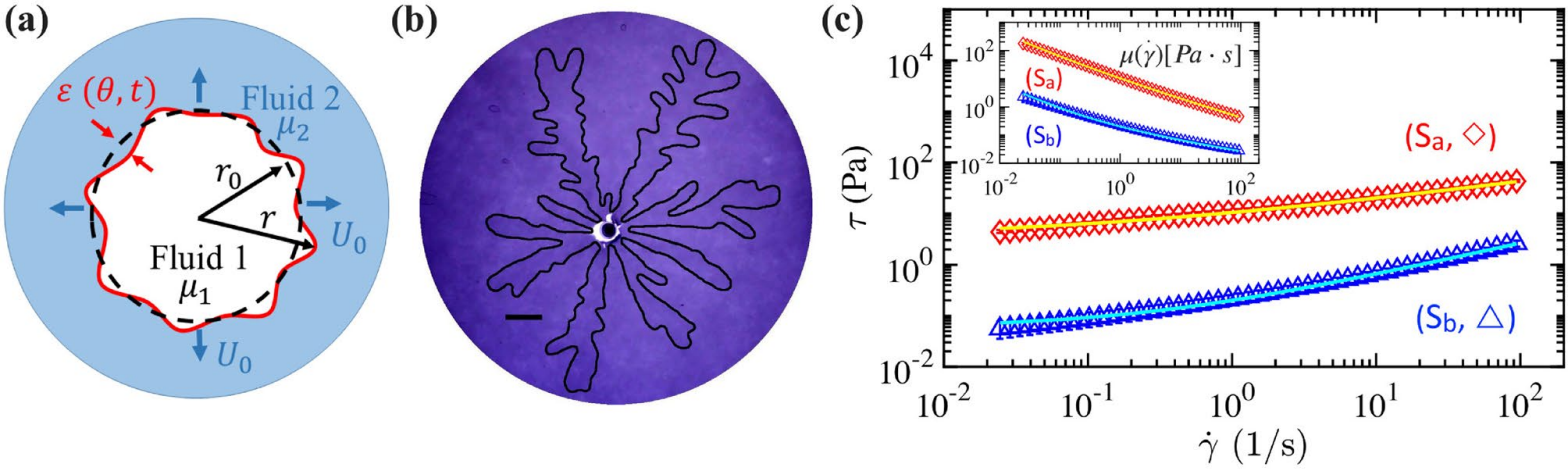
(**a**) Top-view schematics of the fluid-fluid displacement experiment where one less viscous complex fluid 1 of varying viscosity ($\mu _1$) with shear rate (${\dot{\gamma }}$) is pushing another immiscible one, denoted as complex fluid 2 with changing viscosity ($\mu _2$). (**b**) Representative experimental snapshot of complex viscous fingering produced with a complex yield-stress (PAA) solution (S_a_) displaced by a gas injected with a flow rate $Q = 0.2$ slpm (standard liter per minute) in a flat Hele-Shaw cell, under the mobility contrast $\mathcal{M}_c = \mu _2/\mu _1 = 5.58 \times 10^4$ and at the interface velocity $U_0 = 14.3$ mm/s. The scale bar corresponds to 2 cm. (**c**) Flow curves of shear stress, $\tau$, and viscosity, $\mu$, depending on shear rate (${\dot{\gamma }}$) for the two complex solutions used: (S_a_ , ) and (S_b_, ). The lines in (c) correspond to the best fits of the data to the Herschel–Bulkley model (Eq. 1).

Experimentally, we prepare two different aqueous solutions (S_a_ and S_b_) of PolyAcrylic Acid (PAA) to have different viscosity contrasts as the complex displaced fluid (see “Methods”). We first fill in one complex PAA solution in a radial cell and subsequently inject a gas (nitrogen, viscosity $\mu _1 = 1.76 \times 10^{-5}$ Pa $\cdot$ s at 20^∘^C) as a pushing fluid 1 (see Fig. 1a,b). The gas is injected at a constant flow rate, *Q*, ranging from 0.02 to 2 slpm (standard liter per minute) by a flow controller (Alicat) with an accuracy of 1 ml/min. Figure 1c shows the rheological measurements (Anton Paar MCR302) of the shear stress ($\tau$) varying with shear rate (${\dot{\gamma }}$) for the two complex solutions; (S_a_) is more viscous than (S_b_). Neglecting the elastic properties (see “Methods” for the justification), the flow curve data shows an excellent fit with the common Herschel−Bulkley (HB) model^25^:

1 $$\begin{aligned} \tau = \tau _{c} + \kappa {\dot{\gamma }}^{n}, \end{aligned}$$

where $\tau _{c}$, $\kappa$, and *n* correspond to the yield stress, consistensy index, and power-law index, respectively.

The viscosity data ($\mu$) varying with shear rate (${\dot{\gamma }}$) is well described by the corresponding HB model (Eq. 1) via $\mu \equiv \tau /{\dot{\gamma }} = \tau _{c}/{\dot{\gamma }} + \kappa {{\dot{\gamma }}}^{n-1}$, shown in Fig. 1c inset. Table 1 summarizes the best nonlinear-fit results of the rheological measurements of $\tau = f({\dot{\gamma }})$ for $0.025 \le {\dot{\gamma }} \le$ 86 s^-1^. The corresponding HB fitting functions are plotted as lines in Fig. 1c. Both solutions are shear-thinning, with decreasing viscosity with increasing ${\dot{\gamma }}$, i.e., $n < 1$. However, the neutralized PAA solution with NaOH (S_a_) is more viscous, by $\approx ~15\times - 70\times$ than (S_b_) without NaOH depending on ${\dot{\gamma }}$, and has a greater $\tau _c$ but a smaller *n*.

**Table 1:** Rheological parameters for the two complex fluids with the HB model (Eq. 1).

Complex solution	PAA (wt %)	NaOH (wt %)	$\tau _{c}$ (Pa)	$\kappa$ (Pa$\cdot$s^n^)	*n*
(S_a_)	0.10	0.034	3.29	7.12	0.37
(S_b_)	0.10	0	0.06	0.14	0.63

## Results and discussion

Using flat Hele−Shaw cells of a fixed gap thickness, we observe complex fingering patterns, which overall resemble the classical viscous fingering for simple Newtonian fluids but has complex side-fingers along the side of the major fingers, as shown in Fig. 1b. In agreement, similar patterns were observed previously, referred as side-branching^26^ or the elasto-inertial regime^27,28^ in a uniform cell. Interestingly, the side-branched fingers can be obtained only at high flow rates ($Q \ge 1.5$ slpm) for the less viscous fluid (S_b_) but for all the experimental range of $Q = 0.02-1.5$ slpm for the more-viscous fluid (S_a_).

**Figure 2:**
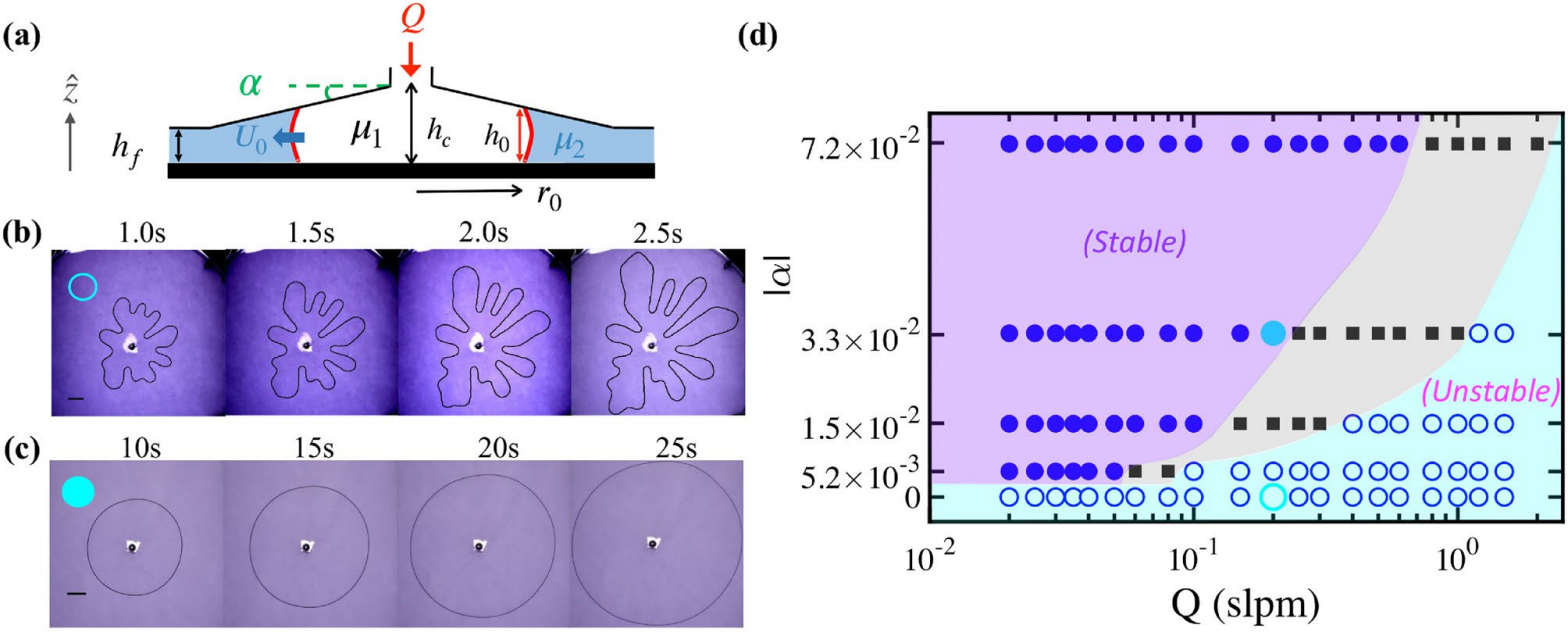
Control of complex viscous fingering using a radially-tapered cell, with a linearly varying gap thickness ($h(r) = h_c + \alpha r$), schematically shown in (**a**), the side-view of the experiment. Here, $h_c$ is the gap-thickness at the cell centre $(r = 0)$. (**b**) Experimental snapshots of a branched viscous fingering pattern observed when a gas is pushing the complex solution (S_b_) in a flat Hele-Shaw cell with $h_c = 0.5$ mm and $Q = 0.2$ slpm (standard liter per minute). (**c**) By contrast, snapshots of a stable interface obtained when the gas is pushing (S_b_) in a tapered cell of the gap gradient $\alpha = -3.33 \times 10^{-2}$, with $h_c = 5.16$ mm and $Q = 0.2$ slpm. The scale bars in (b) and (c) represent 20 mm. (**d**) Experimental results of stability diagram, with uniform stable (filled circle) versus fingering/wavy unstable interfaces (open circle) under various values of flow rate, *Q*, and the tapered gap gradient, $|\alpha |$. Black squares ($\blacksquare$) represent a transitional state where the interface starts to develop a wavy profile.

When using a radially converging cell of a constant and negative gap-thickness gradient, $\alpha = dh(r)/dr$ (see Fig. 2a), we stopped observing side-branched fingers but smooth classical viscous fingers for more-viscous (S_a_) with using Q = $0.02-1.5$ slpm. This is consistent with a recent experimental study^29^, revealing mitigation of side-branching, but not inhibition of the primary viscous fingering, for a complex yield-stress fluid in a rectangular tapered cell. Is it even possible to eliminate complex viscous fingerings for a unfavorable mobility contrast, $\mathcal{{M}}_c \sim \mu _2/\mu _1 > 1$? To answer this critical question, we systematically conduct experiments of a gas pushing a PAA complex fluid under various flow rates (*Q*) and five different taper gradients ($\alpha$). Using a different complex solution, remarkably, with suitable rheological and flow parameters, we can control and inhibit the primary fingering instability and observe complete stable and flat interfaces between the pushing Newtonian gas and the displaced, complex fluid (S_b_) (of $\mathcal{M}_c \approx 10^3 - 10^5$), as illustrated by a fully circular displacement front, i.e., stable interface, in Fig. 2c.

Figure 2d shows the experimental phase diagram of unstable versus stable displacements manifested in wavy fingering (e.g., Fig. 2b) or a smooth circular (e.g., Fig. 2c) interfacial pattern, when the gas is pushing the complex fluid (S_b_) under various values of $\alpha$ and *Q*. We differentiate three types of displacements observed during the experiments with the fluid (S_b_), namely uniform stable (filled circle, ), fingering/wavy unstable (open circle, ), and transitional (filled square, ) displacements. The latter corresponds to the transitional state where the interface starts to develop a wavy profile.

A crucial observation from Fig. 2d is that, firstly, a stable interface accompanying with a full displacement occurs at low *Q* in a converging-gap cell, whereas an unstable interface with fingering pattern always happen in a uniform-gap cell (of $\alpha = 0$). Secondly, a steeper converging gap gradient helps stabilize the interface, and the transition from stable to unstable interfaces happens at a higher flow rate *Q* as the gap-gradient value $|\alpha |$ is increased. Thirdly, such experimental stability diagram is established only for the complex fluid (S_b_) since a complete sweep has never been observed with our experimental parameters for the more-viscous fluid (S_a_) of a high mobility contrast, $\mathcal{M}_c = \mu _2/\mu _1$ = $2.68\times 10^{4} - 1.16\times 10^{7}$. The complex fluid (S_b_) has a lower and small $\mathcal{M}_c$-range of $1.61\times 10^{3} - 1.67\times 10^{5}$ than (S_a_). These contrast results between the two complex solutions highlight not only the complexity but also the importance of rheological parameters, via $\kappa$, *n* and local ${\dot{\gamma }}$, in controlling complex viscous fingering.

To gain physical insights, we develop a simplified linear stability analysis generalized to two yield-stress, power-law fluids (Fluid 1 pushing Fluid 2) in a radially-tapered cell, as depicted in Fig. 2a. The introduction of a constant gap gradient ($\alpha$) produces a linearly-varying height, *h*(*r*), between the two plates of the cell so that $h(r) = h_c + \alpha r$, where $h_c$ is the gap-thickness at the cell centre $(r = 0)$. Considering the fluids’ interface at $r = r_0$, the height can be expressed as $h(r) = h_0 + \alpha (r-r_0)$, with $h_0$ the gap-thickness at the fluid-fluid interface. For fluids in the narrow gap, we use a modified Darcy’s law replacing the constant viscosity ($\mu$), typically applicable for a simple Newtonian fluid, by the effective shear-dependent viscosity, $\mu _\text {eff}$, to approximate the complex fluid’s velocity in a homogeneous porous medium. Although this model may not be rigorously accurate for all types of non-Newtonian fluids in different flow regimes^30,31^, the applicability of this approximation has been verified numerically for a Bingham fluid at low-pressure, one-channel and high-pressure, fully flowing regimes^31^ and validated experimentally for weakly shear-thinning fluids^32^ and for yield-stress gel solution (of shear-thinning [$n \approx 0.4$] and with a yield stress ranging from 10 to 50 Pa)^26^. Since our complex fluids, S_a_ and S_b_, are shear-thinning with less important values of yield-stress, the applicability of the modified Darcy’s law describing our complex fluids’ velocity field may be expected and allows us to arrive at an analytical solution (given below).

Neglecting the fluids’ elastic properties (justified in the Section of “Methods”), the governing equations of the immiscible, complex fluids are the continuity equation (taking gap-variation into account) and 2D depth-average modified Darcy’s law using $\mu _\text {eff}$:

2 $$\begin{aligned} \nabla \cdot \left( h \textbf{U}_j \right) = 0 \quad \text {and} \quad \textbf{U}_j = -\frac{h^2}{12\mu _{\text {eff}j}} \vec {\nabla } P_j, \end{aligned}$$

where $\textbf{U}_j(r,\theta ) = (u_{rj}, u_{\theta j})$ and $P_j(r,\theta )$ are the depth-average velocity and pressure fields of the fluid indexed *j*, respectively. *j* represents the two complex fluids during the fluid-fluid displacement process; $j = 1$ (2) denotes the pushing (displaced) fluid.

The complex fluid’s viscosity ($\mu _{\text {eff} j}$) is modeled using the Herschel–Bulkley law (see Eq. 1) for yield-stress fluids, with the local shear rate approximated by ${\dot{\gamma }}(r) = u_{rj}/h(r)$, and can be expressed as: $\mu _{\text {eff} j} = \frac{\tau _{cj}}{\overset{.}{\gamma }} + \kappa _j {\overset{.}{\gamma }}^{n_j-1}$, with yield stress ($\tau _{cj}$), consistensy index ($\kappa _j$), and power-law index ($n_j$). The depth-average continuity equation can be expressed using the pressure field ($P_j$) and further simplified. By setting $n_j = 1$ and $\tau _{cj} = 0$, we obtain and recover the simple Newtonian fluid case: $\frac{\partial ^2 P_j}{\partial r^2} + \frac{1}{r} \frac{\partial P_j}{\partial r} + \frac{3 \alpha }{h} \frac{\partial P_j}{\partial r} + \frac{1}{r^2} \frac{\partial ^2 P_j}{\partial \theta ^2} = 0$^22^.

In the linear stability analysis, the pressure field is expressed as the solutions of the base state and the perturbation, $\epsilon (\theta ,t) = \epsilon _0 r_0(t) \exp { \left( i k \theta + \sigma t \right) }$:

3 $$\begin{aligned} P_j (r,\theta ,t) = f_j (r) + g_{kj} (r) \epsilon (\theta ,t), \end{aligned}$$

where $f_j(r)$ corresponds to the base-state pressure when the interface is stable and independent of $\theta$. The term of $g_{kj}(r) \epsilon$ represents the perturbation that propagates along the interface with wavenumber, *k*, and the growth rate of the perturbation, $\sigma$. We employ the kinematic boundary conditions, i.e., two complex fluids moving at the same velocity at the interface, and the Young−Laplace equation for the pressure jump at the interface due to surface tension and curvature. To obtain analytical solutions, we assume that the fluid yield stress is negligible compared to the viscous stress, i.e., small Bingham ($Bn_j \ll 1$) situation, where the Bingham number is the ratio of the yield to viscous stress: $Bn_j= \frac{\tau _{cj}}{\kappa _j \left( \frac{u_{rj}}{h} \right) ^{n_j}}$. Focusing on the moment when the perturbation starts to propagate, implying small perturbation $\left( \epsilon \ll 1 \right)$, $\epsilon _0 \ll 1$, $g'_{kj}(r) \epsilon \ll f'_j(r)$, and negligible high-order terms of $O \left( \epsilon ^2 \right)$, we obtain the dimensionless dispersion relation Eq. (4) below, with the dimensionless growth rate, ${\bar{\sigma }} = \frac{\sigma r_0}{U_0}$, and the dimensionless wavenumber, ${\bar{k}} = k$ (see Supplementary Information for the detailed derivation):

4 $$\begin{aligned} \frac{12 {\overline{\sigma }} h_0}{\gamma } \left( \kappa _1 \sqrt{n_1} \left( \frac{U_0}{h_0} \right) ^{n_1} + \kappa _2 \sqrt{n_2} \left( \frac{U_0}{h_0} \right) ^{n_2} \right)&= -\frac{12 U_0}{\gamma } \left( \sqrt{n_1} \mu _1|_{r=r_0} + \sqrt{n_2} \mu _2|_{r=r_0} \right) \nonumber \\&\quad - \frac{12 \alpha r_0}{\gamma } \biggl ( 2 \sqrt{n_1} \tau _{c1} + 2 \sqrt{n_2} \tau _{c2} \biggl ) \nonumber \\&\quad - \frac{12 \alpha r_0}{\gamma } \biggl ( \kappa _1 \sqrt{n_1} \left( \frac{U_0}{h_0} \right) ^{n_1} + \kappa _2 \sqrt{n_2} \left( \frac{U_0}{h_0} \right) ^{n_2} \biggl )\nonumber \\&\quad + {\overline{k}} \left( \frac{12 U_0}{\gamma } \left( \mu _2|_{r=r_0} - \mu _1|_{r=r_0} \right) + 2 \alpha \cos {\theta _c} + \frac{{h_0}^2}{{r_0}^2} \right) - \frac{{h_0}^2}{{r_0}^2} {{\overline{k}}}^3, \end{aligned}$$

where $\gamma$ is the interfacial tension, the complex fluids’ viscosity at the interface is given as $\mu _j|_{r = r_0} = \left( \tau _{cj} + \kappa _j \left( \frac{U_0}{h_0}\right) ^{n_j} \right) \frac{h_0}{U_0}$, and $\theta _c$ corresponds to the contact angle at the interface and is measured between the plate and the curved meniscus (across the gap). $\theta _c$ = 0 corresponds to a completely wetting displaced fluid, whereas $\theta _c = \pi$ to a perfectly non-wetting one.

Consequently, taking $\tau _{cj} =0$, $n_j = 1$, $\kappa _j = \mu _j$ in Eq. (4) for simple fluids, and defining the viscosity contrast $\lambda = \frac{\mu _1}{\mu _2}$ and Capillary number $Ca=\frac{12 U_0 \mu _2}{\gamma }$, the dispersion relation recovers to the same formula by Al-Housseiny and Stone for Newtonian fluids with constant viscosity (Eq. 24 in Ref.^22^). In addition to the crucial influences of $\alpha$, $\lambda$, *Ca*, and wetting angle ($\theta _c$) for the simple fluid case, the derived dimensionless perturbation growth rate (${\overline{\sigma }}$) as a function of (${\overline{k}}$) depends on the fluids’ rheological properties ($\kappa _j, n_{j}, \tau _{cj}$) and the local velocity, radius, and gap thickness at the interface ($U_0, r_0, h_0$, respectively) for the complex yield-stress fluids. The primary stabilizing mechanism for both Newtonian^20,21,23^ and Non-Newtonian viscous fingering instabilities using a converging taper is similar. That is through a stabilizing Capillary effect, via the term of $2 \alpha \cos {\theta _c}$ in Eq. (4) as $\alpha < 0$, since the Laplace pressure becomes more significant when the fluid-fluid interface travels across a narrower gap and can overcome the destabilizing effect of the pressure gradient due to viscosity difference, via the term $\frac{12 U_0}{\gamma } (\mu _2|_{r=r_0} - \mu _1|_{r=r_0})$. However, comparing the expression of the dimensionless dispersion relation (Eq. 4) to the Newtonian counterpart (in Ref.^22^), we notice an additional, distinct term via $-\frac{12\alpha r_0}{\gamma } ( \sqrt{n_1} \tau _{c1} + \sqrt{n_2} \tau _{c2})$, which has a positive value for a converging taper since $\alpha < 0$, while $r_0$, $\gamma$, $n_j$, and $\tau _{cj}$ have positive values. This positive term, depending on the yield stress of the fluids, tends to increase the growth rate and, hence, has a destabilizing effect promoting the fingering instability growth, which could explain the more likely appearance of viscous fingering using complex fluids. Additionally, instead of being constant in the Newtonian case, the viscosity value depends on various factors, such as the interfacial location, thickness, and velocity as well as the rheological valuables of $\tau _c$, *k*, and *n*. On the one hand, if the pertubation’s growth rate *σ* is less than zero for every wavenumber, *k*, the interface will always be stable theoretically. On the other hand, the wavenumber at the maximum growth (${\bar{k}}_{max}$) can be found by taking the derivative of the above dimensionless dispersion Eq. (4) with respect to ${\bar{k}}$ and setting $\frac{ \partial {\overline{\sigma }}}{\partial {\overline{k}}} = 0$:

5 $$\begin{aligned} {\bar{k}}_{max} = \left( \frac{\frac{{h_0}^2}{{r_0}^2} + 2 \alpha \cos {\theta _c} + \frac{12 U_0}{\gamma } \left( \mu _2|_{r=r_0} - \mu _1|_{r=r_0} \right) }{3 \frac{{h_0}^2}{{r_0}^2}} \right) ^{\frac{1}{2}}. \end{aligned}$$

${\bar{k}}_{max}$ is the wave number at the maximum growth, i.e., theoretically, the most unstable mode’s wave number. Hence, $\sigma ({\bar{k}}_{max}) < 0$ theoretically signifies the stability of the interface, that is, no growth of the most unstable mode in this situation.

**Figure 3:**
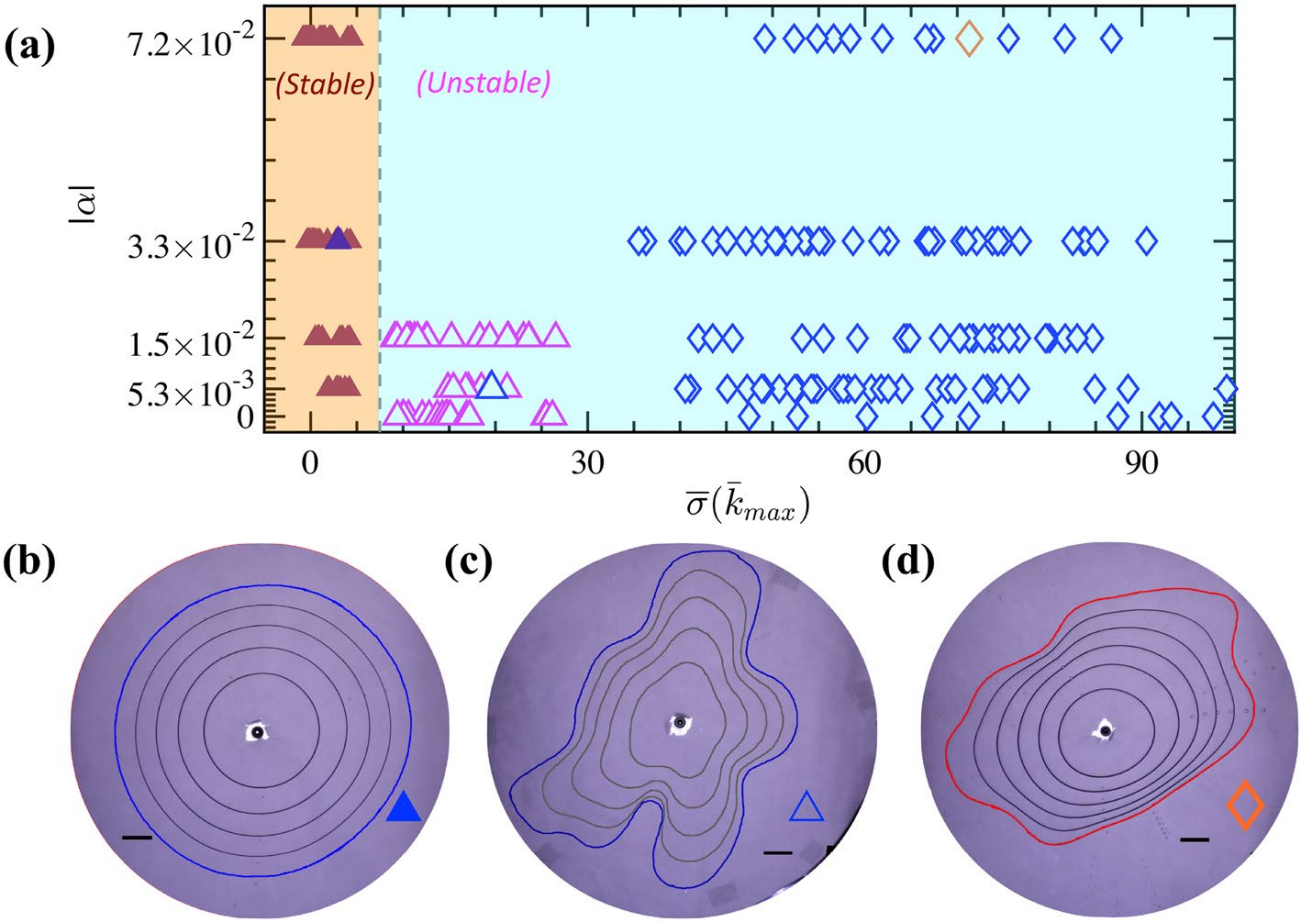
Comparison between experimental and theoretical results: (**a**) The growth rate of the perturbation at the most unstable mode of $k_{max}$, ${\overline{\sigma }}({\bar{k}}_{max})$ using Eqs. (4) and (5) for different experiments performed with various gap-gradient, $|\alpha |$. The experiments with the more viscous solution (S_a_) always show unstable wavy interface (). In contrast, for the less viscous complex fluid (S_b_), stable displacement () and unstable interface () are observed with nearly-zero and relatively-large growth rate ${\overline{\sigma }}$, respectively. Theoretically, stable interfaces occur with a negative growth rate, i.e., when ${\overline{\sigma }} < 0$. Consistently, our experiments show stable displacements (filled symbols) when ${\overline{\sigma }} < 7.5$. (**b**)–(**d**) are the overlays of experimental snapshots, revealing the evolution of the interface profiles for the three big symbols (, , ) in (a), respectively. The time steps are $\delta t=22$ s, 0.6 s, and 1 s in (b), (c) and (d), respectively. Each scale-bar represents a length scale of 20 mm.

It turns out that the expression of ${\bar{k}}_{max}$ for the PAA complex fluid is rather similar to its Newtonian counterpart. Similar to the Newtonian scenario, one noticeable result from Eq. (5) is that ${\bar{k}}_{max}$ primarily varies with the interfacial velocity $U_0$, which is increased with *Q* and (slightly) decreased with increasing $|\alpha |$ for a fixed $h_f$, the taper’s thickness at the edge (see Fig. 2a). In addition, a converging gap gradient ($\alpha <0$) helps reduce the value of ${\bar{k}}_{max}$, while an important difference in viscosity, via the local term of $\mu _2 - \mu _1$, has a destabilizing effect on the interface, enlarging the values of ${\bar{k}}_{max}$. A significant difference compared to the Newtonian case is that the viscosity terms now encompass the effect of complex fluids’ yield-stress $\tau _c$ and other rheological parameters through *k* and *n*.

Using the wavenumber of maximum growth ${\bar{k}}_{max}$ (Eq. 5) and the dimensionless dispersion relation (Eq. 4), we obtain the growth rate at the most unstable mode, ${\overline{\sigma }}({\bar{k}}_{max})$. To compare with our theoretical prediction, we plotted in Fig. 3a the values of ${\overline{\sigma }}({\bar{k}}_{max})$ using Eq. (4), with the values of viscosity ($\mu _1$ and $\mu _2$) and the velocity, location, and gap-thickness at propagating fluid-fluid interface ($U_0$, $r_0$ and $h_0$) analyzed from the experiments. From Fig. 3a, we can observe a transition from stable (filled symbols) to unstable (open symbols) interfaces when ${\overline{\sigma }}({\bar{k}}_{max}) < 7.5$ from the experimental data, slightly deviating from the theoretical value of ${\overline{\sigma }} < 0$ for a stable interface with a decay growth rate. In other words, the experimental values show a bound growth rate, i.e., a limited growth rate for the instability, while the experimental interface is still stable in reality. Figure 3b–d are the overlays of representative experimental snapshots, revealing the evolution of the interface profiles of stable (filled blue triangle) and unstable displacement (open blue triangle) for (S_b_) while unstable interface (open orange diamond) for (S_a_).

The deviation between our experimental results and theoretical prediction of the stable versus unstable displacement may be due to the few assumptions we made. For example, the impact of the gravity and the elastic properties have been ignored. Moreover, whenever $|\alpha |$ is getting bigger, the assumptions of small ratio of gap change ($\frac{\alpha (r-r_0)}{h_0} \ll 1$) as well as the characteristic length scale over which the depths varies being much larger than that of the perturbation scale ($\frac{k h_0}{\alpha r_0} \gg 1$) may not be applicable. Last but not the least, we neglected the yield stress compared to the viscous stress by assuming small $Bn \ll 1$. These assumptions likely contribute to the drifting of the critical growth rate at the most unstable mode from 0 (theoretically) to 7.5 (observed with our experimental conditions).

## Conclusions

In summary, we have demonstrated a powerful way of stabilizing the primary viscous fingering instability for complex yield-stress, power-law fluids using a tapered narrow cell experimentally and theoretically for the first time. Experimentally, using a radially converging taper, we can hinder complex fingering patterns, e.g., eliminating side-fingers for the more-viscous (S_a_) of $M_c \approx 10^4 - 10^7$ and suppressing wavy interfaces completely for the less-viscous complex fluid (S_b_) of $M_c \approx 10^3 - 10^5$. With a linear stability analysis using the simplified, effective Darcy’s law, we derive the dispersion relation and establish a convenient stability criterion corresponding to the perturbation’s growth rate of the most unstable mode. In addition to the viscosity contrast ($\lambda = \mu _1/\mu _2$), gap gradient ($\alpha$), $\theta _c$ and Capillary number (*Ca*) for the simple Newtonian fluids, several vital parameters affect the complex fluids’ viscous fingering stability criterion, namely the fluid’s rheological characteristics, such as $\kappa$, $\tau _c$, *n*, and $\gamma$, as well as the interface position, gap thickness, and velocity ($r_0$, $h_0$ and $U_0$, respectively). Firstly, these parameters affects the value of the wavenumber of maximum growth $k_{max}$ and the dispersion relation via the complex expression of the local viscosity for yield-stress fluids. Secondly, compared to the Newtonian dispersion relation, an additional (positive) term—depending on the yield-stress and power-law index of the fluids—appears in Eq. (4) for a converging taper, thereby destabilizing the interface and promoting fingering pattern for the complex fluids. This theoretical stability criterion through ${\overline{\sigma }}({\bar{k}}_{max})$, despite the assumption of small $Bn \ll 1$, shows fair agreement with the experimental results using two low yield-stress fluids of distinct mobility ratios. These results, particularly the complex dispersion relation (Eq. 4) and ${\overline{\sigma }}({\bar{k}}_{max})$, provide quantitative insights into the designs and strategies for controlling viscous fingering and interfacial profiles during complex fluids’ displacement in microfluidics, narrow cells, packed beads, and porous media.

## Methods

### Sample preparation

The two aqueous solutions of PAA (SigmaAldrich, molecular weight: $M_w \approx 1,250,000$) are prepared to produce different viscosity contrasts. Both solutions have the same polymer concentration, by slowly adding the polymer powder in water and subsequently stirring the mixture at high speed for 1 hr. The mixture generates an acid solution that can be neutralized using a basic solution. The two PAA solutions: (S_a_) and (S_b_) are prepared with and without NaOH (S_b_), respectively, by stirring them for another 10 hours at medium speed. Finally, after the agitation, the solution is allowed to rest for a day before performing rheological measurements.

### Rheological measurements

**Figure 4:**
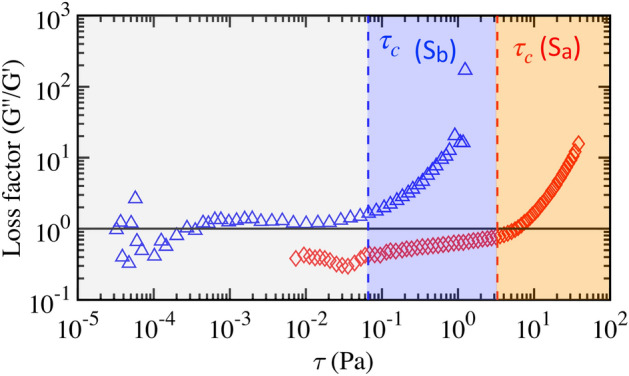
The data of loss factor, the ratio of the loss modulus ($G''$) to the storage modulus ($G'$), varying with the shear stress, $\tau$, obtained during oscillation amplitude sweep test at constant frequency (${\hat{w}} = 1$ rad/s). The vertical dashed lines represent the yield-stress ($\tau _c$) values of the two fluids.

We further perform oscillation amplitude sweep tests at constant frequency (${\hat{\omega }}$ = 1 rad/s) to validate negligible elasticity of the complex fluids. Shown in Fig. 4 below are the results of the loss factor, i.e., the ratio of the loss modulus ($G''$) to the storage modulus ($G'$). The former $G''$ represents the viscous properties of the complex fluids, while the latter $G'$ fluid elasticity with respect to the shear stress, $\tau$. The fluid’s viscous behavior prevails when the loss factor ($G''/G'$) is greater than unity, whereas elastic for $G''/G' < 1$. The vertical dashed lines represents the yield-stress values for the fluids (S_a_) and (S_b_). We only focus on the (color shaded) regime whereby fluids are flowing, i.e, $\tau > \tau _c$, when $G''/G' \gtrsim 1$, meaning the viscous component prevails. Hence, we can neglect the elastic effects of the fluids in the theoretical analysis.

### Supplementary Information

Supplementary Information.

## Data Availability

Authors can confirm that all relevant data are included in the paper and/or its supplementary information files.
