# Supplementary material for: Controlling viscous fingering instabilities of complex fluids
Source: Sci Rep. 2024 Jan 29;14:2338. doi: 10.1038/s41598-024-52218-w (PMC10822875; doi:10.1038/s41598-024-52218-w)
Supplement: Supplementary file 1 — Supplementary Information. [file 41598_2024_52218_MOESM1_ESM.pdf]

Supporting Information for
"Controlling Viscous Fingering Instabilities of Complex Fluids"

Alban Pouplard[1] and Peichun Amy Tsai[1,†]

[1] *Department of Mechanical Engineering,*
University of Alberta, Edmonton, Alberta, Canada T6G 1H9

Abstract

In this supplementary material, we include the detailed deviation of the linear stability analysis.

† Corresponding author: peichun.amy.tsai@ualberta.ca

1. LINEAR STABILITY ANALYSIS

We develop a linear stability analysis generalized to both yield-stress, power-law complex fluids (Fluid 1 pushing Fluid 2) in a radially tapered cell (see Fig. 2a in the main article). The introduction of a constant gap gradient (α) produces a linearly-varying height between the two plates of the cell. Considering the fluids' interface at $r = r_0$, the height $h(r)$ can be expressed as $h(r) = h_0 + \alpha(r - r_0)$, with h_0 the gap thickness at the interface.

In the theoretical derivation, we use a modified Darcy's law replacing the constant viscosity, μ, by an effective shear-dependent viscosity, μ_{eff}. This approach has been used in other derivations to model non-Newtonian flow in a homogeneous porous medium or a uniform Hele-Shaw cell [1–4]. Although this model may not be rigorously accurate for all types of non-Newtonian fluids [5, 6], the applicability of this approximation has been verified numerically for Bingham fluid at low-pressure, one-channel and high-pressure, fully flowing regimes [6] and theoretically for weakly shear-thinning fluids [5, 7]. Furthermore, such applicability has been experimentally validated for weakly shear-thinning fluids [4], for a yield-stress (oil-in-water) emulsion [8], and for a yield-stress gel solution (of shear-thinning [$n \approx 0.4$] and with a yield stress ranging from 10 to 50 Pa) [9]. Since our complex fluids $\mathrm{S_a}$ and $\mathrm{S_b}$ are shear-thinning with less important values of yield-stress, the applicability of the modified Darcy's law describing our complex fluids' velocity field can be expected and allows us to arrive at an analytical solution (given below).

Neglecting the fluid's elastic properties [10], which is justified for our experimental case, the governing equations are the continuity equation and 2D depth-average Darcy's law:

$$\nabla \cdot (h\mathbf{U}_j) = 0, \tag{S1}$$

$$\mathbf{U}_j = -\frac{h^2}{12\mu_{\text{eff}j}}\vec{\nabla}P_j. \tag{S2}$$

$\mathbf{U}_j(r,\theta) = (u_{rj}, u_{\theta j})$ and $P_j(r,\theta)$ are the depth-average velocity and pressure fields of the fluid indexed j, respectively. j represents the two complex fluids during the fluid-fluid displacement process; $j = 1$ (2) denotes the pushing (displaced) complex fluid.

The complex fluid's viscosity ($\mu_{\text{eff}j}$) is modeled using the Herschel–Bulkley law [11] for yield-stress fluids, with the local shear rate approximated via $\dot{\gamma} = u_{rj}/h(r)$, and expressed as:

$$\mu_{\text{eff}j} = \frac{\tau_{cj}}{\dot{\gamma}} + \kappa_j \dot{\gamma}^{n_j - 1}, \tag{S3}$$

where τ_{cj}, κ_j and n_j correspond to the yield stress, the consistency index, and the power-law index, respectively.

By defining the Bingham number as the ratio of the yield to viscous stress: $Bn_j = \frac{\tau_{cj}}{\kappa_j\left(\frac{u_{rj}}{h}\right)^{n_j}}$, we express $\frac{\tau_{cj}}{\frac{h}{12}\frac{\partial P_j}{\partial r}} = -\frac{1}{1+\frac{1}{Bn_j}}$. The cell's gap thickness can be expressed as $h = h_0\left(1+\frac{\alpha(r-r_0)}{h_0}\right)$. We further assume a small ratio of gap change, i.e., $\frac{\alpha(r-r_0)}{h_0} \ll 1$. With Eq. (S2)–(S3), the depth-average continuity Eq. (S1) can be expressed using pressure field (P_j) and simplified into:

$$\frac{\partial^2 P_j}{\partial r^2} + \frac{n_j}{r}\frac{\partial P_j}{\partial r} + \frac{(2n_j+1)\,\alpha}{h_0}\frac{\partial P_j}{\partial r} + \frac{12n_j\alpha\tau_{cj}}{h_0^2} + \frac{12n_j\tau_{cj}}{h_0 r} + \frac{12n_j\tau_{cj}\alpha}{{h_0}^2}\frac{r_0}{r} + \frac{n_j}{r^2}\frac{\partial^2 P_j}{\partial\theta^2}\left(1-\frac{1}{1+\frac{1}{Bn}}\right) + \frac{1}{r^2}\frac{\partial P_j}{\partial\theta}\left[(1-n_j)\frac{\frac{\partial^2 P_j}{\partial\theta\partial r}}{\frac{\partial P_j}{\partial r}} - n_j\frac{\tau_{cj}\frac{\partial^2 P_j}{\partial\theta\partial r}}{\frac{h}{12}\left(\frac{\partial P_j}{\partial r}\right)^2}\right] = 0. \tag{S4}$$

By setting $n_j = 1$ and $\tau_{cj} = 0$, the above Eq. recovers to the simple Newtonian fluid case: $\frac{\partial^2 P_j}{\partial r^2} + \frac{1}{r}\frac{\partial P_j}{\partial r} + \frac{3\alpha}{h}\frac{\partial P_j}{\partial r} + \frac{1}{r^2}\frac{\partial^2 P_j}{\partial\theta^2} = 0$, reported by Al-Housseiny and Stone [12].

In the linear stability analysis, the pressure field is expressed as the solutions of the base state and the perturbation, $\epsilon(\theta,t) = \epsilon_0 r_0(t)\exp(ik\theta+\sigma t)$:

$$P_j(r,\theta,t) = f_j(r) + g_{kj}(r)\epsilon(\theta,t). \tag{S5}$$

Here, $f_j(r)$ corresponds to the base-state pressure when the interface is stable and independent of θ. The term of $g_{kj}(r)\epsilon$ represents the perturbation that propagates along the interface, with wavenumber (k) and the growth rate of the perturbation (σ).

We focus at the moment when the perturbation starts to propagate, implying small perturbation ($\epsilon \ll 1$), $g'_{kj}(r)\epsilon \ll f'_j(r)$, and negligible high-order terms of $O(\epsilon^2)$. Since we are interested in the solution of P_j in the vicinity of the perturbed interface, we can linearize around the base state such that: $r = r_0(1+\epsilon_0 z)$ with $\epsilon_0 \ll 1$. Substituting Eq. (S5) into Eq. (S4), using $\epsilon_0 \ll 1$ to linearize terms and neglecting $O({\epsilon_0}^2)$ but not $O({\epsilon_0}^2k^2)$, the solutions of the base and perturbed states can be found with the following Eqns:

$$\frac{d^2 f_j(z)}{\partial z^2} + n_j\epsilon_0\frac{df_j(z)}{dz} + \frac{(2n_j+1)\,\alpha}{h_0}\epsilon_0 r_0\frac{df_j(z)}{dz} = 0. \tag{S6}$$

$$\frac{d^2 g_{kj}(z)}{dz^2} + n_j\epsilon_0\frac{dg_{kj}(z)}{dz} + \frac{(2n_j+1)\,\alpha}{h_0}\epsilon_0 r_0\frac{dg_{kj}(z)}{dz} - n_j k^2{\epsilon_0}^2 g_{kj}\left(1-\frac{1}{1+\frac{1}{Bn_j}}\right) = 0. \tag{S7}$$

At $r = r_0$, from the Darcy's law and with the fact that $g'_{kj}(r)\epsilon \ll f'_j(r)$, we solve for the specific solution for the base-state pressure, $f_j(r)$, expressed with $z = \frac{r-r_0}{\epsilon_0 r_0}$:

$$f_j(r) = F_j \exp\left(-\left(n_j + \frac{(2n_j+1)\,\alpha r_0}{h_0}\right)\frac{r-r_0}{r_0}\right),$$
$$F_j = \frac{12\left[\tau_{cj} + \kappa_j\left(\frac{U_0}{h_0}\right)^{n_j}\right]}{h_0}\frac{r_0}{n_j + \frac{(2n_j+1)\alpha r_0}{h_0}}. \tag{S8}$$

To solve the perturbation Eq. (S7) analytically, we assume $(1+\frac{1}{Bn_j})^{-1} \ll 1$, equivalent to small $Bn_j(\ll 1)$ situation when the yield stress is negligible compared to the viscous stress. We assume that the length scale of the interfacial perturbation (i.e, $|\frac{r_0}{k}|$) is much smaller than the length scale over which the depth varies scaling as $|\frac{h_0}{\alpha}|$. Under this assumption, we have $|\frac{\alpha r_0}{kh_0}| = O(\epsilon)$ as h_0 scales as $|\alpha r_0|$. We then obtain a simplified solution for a small Bn_j $(\ll 1)$:

$$g_{kj}(z) = B_{kj}\exp(r_{kj+}z) + C_{kj}\exp(r_{kj-}z),$$
$$r_{kj+} = -\epsilon_0\sqrt{n_j}k, \quad r_{kj-} = \epsilon_0\sqrt{n_j}k.$$

Moreover, it is physically impossible for the perturbation to grow in space from its origin. This means that for a fluid indexed 1 pushing a fluid indexed 2, we have the following constraints: $g_{k1}|_{r\to 0} = 0$, $g_{k2}|_{r\to+\infty} = 0$, $g_{k1}|_{z\to-\infty} = 0$, $g_{k2}|_{z\to+\infty} = 0$. As a consequence, the solution can be expressed with $z = \frac{r-r_0}{r_0\epsilon_0}$:

$$g_{kj} = B_{kj}\exp\left(m_{kj}\frac{r-r_0}{r_0}\right), \tag{S9}$$
$$m_{kj} = (-1)^{j+1}\sqrt{n_j}k. \tag{S10}$$

By linearizing the exponential terms in Eq. (S8)-(S9), we can evaluate the pressure at the interface ($r_{\text{int}} = r_0 + \epsilon$):

$$P_j|_{r=r_0+\epsilon} = \frac{12\left[\tau_{cj} + \kappa_j\left(\frac{U_0}{h_0}\right)^{n_j}\right]}{h_0}\frac{r_0}{n_j + \frac{(2n_j+1)\alpha r_0}{h_0}} - \frac{12\left(\tau_{cj} + \kappa_j\left(\frac{U_0}{h_0}\right)^{n_j}\right)}{h_0}\epsilon + B_{kj}\epsilon + O\left(\epsilon^2\right). \tag{S11}$$

To determine the coefficient B_{kj}, we use the kinematic boundary condition (i.e., the same fluid velocity) at the interface for $r = r_{\text{int}} = r_0 + \epsilon$, i.e. $\frac{\partial r_{\text{int}}}{\partial t} = u_{rj}|_{r=r_0+\epsilon}$. By linearizing $\frac{\partial P_j}{\partial r}$ at the interface, using the assumptions of $U_0 \gg \sigma\epsilon$ and $\frac{\alpha\epsilon}{h_0} \ll 1$, but neglecting $O(\epsilon^2)$, we find

the expression for B_{kj}:

$$B_{kj} = -\frac{12\kappa_j}{m_{kj}{h_0}^{n_j+1}}\left(n_j\sigma {U_0}^{n_j-1} r_0 + n_j {U_0}^{n_j}\frac{\alpha r_0}{h_0} + n_j\frac{{h_0}^{n_j}\tau_{cj}}{\kappa_j} + 2n_j\frac{{h_0}^{n_j}\tau_{cj}}{\kappa_j}\frac{\alpha r_0}{h_0} + n_j {U_0}^{n_j}\right). \quad \text{(S12)}$$

For simple Newtonian fluids as $n_j = 1$ and $\kappa_j = \mu_j$, we find the same expression for $B_{kj} = -\frac{12\mu_j U_0}{m_{kj}{h_0}^2}\left(\frac{\sigma r_0}{U_0} + \frac{\alpha r_0}{h_0} + 1\right)$ as the solution found previously by Al-Housseiny and Stone [12].

The Capillary pressure jump at the interface is described by the Young-Laplace equation, which accounts for both the lateral curvature (Ψ) and the curvature because of the cell depth $\left(\frac{1}{h}\right)$. However, we will neglect the viscous stresses. At the interface, $r = r_0 + \epsilon(\theta, t)$, for a fluid indexed 1 pushing a fluid indexed 2, the pressure difference across the interface:

$$P_1 - P_2 = \frac{2\gamma\cos\theta_c}{h_0 + \alpha\epsilon} + \gamma\Psi, \quad \text{(S13)}$$

$$\Psi = \frac{r^2 + 2\left(\frac{\partial r}{\partial\theta}\right)^2 - r\frac{\partial^2 r}{\partial\theta^2}}{\left(r^2 + \left(\frac{\partial r}{\partial\theta}\right)^2\right)^{\frac{3}{2}}}, \quad \text{(S14)}$$

where γ is the interfacial tension, and θ_c corresponds to the contact angle at the interface and is measured between the plate and the curved meniscus (across the gap). $\theta_c = 0$ corresponds to a completely wetting displaced fluid, whereas $\theta_c = \pi$ to a perfectly non-wetting one.

Since ${r_0}^2 \gg \epsilon^2$, using the linearization around r_0, and neglecting $O\left(\epsilon^2\right)$, the second term in Eq. (S13) can be simplified to:

$$\gamma\Psi = \gamma\left(\frac{1}{r_0} - \frac{\epsilon}{{r_0}^2} + \frac{k^2\epsilon}{{r_0}^2}\right).$$

Finally, the pressure jump at the interface $r = r_0 + \epsilon(\theta, t)$ can be expressed by:

$$P_1 - P_2 = \frac{2\gamma\cos\theta_c}{h_0} + \frac{\gamma}{r_0} + \gamma\epsilon\left(\frac{k^2 - 1}{{r_0}^2} - \frac{2\alpha\cos\theta_c}{{h_0}^2}\right) + O\left(\epsilon^2\right). \quad \text{(S15)}$$

The terms $\frac{2\gamma\cos\theta_c}{h_0} + \frac{\gamma}{r_0}$ corresponds to the base state; that is the pressure difference at the interface between the two fluids where the interface is stable. The rest are the additional Laplace pressure due to the perturbation at the interface.

We substitute the expression of the linearized pressure Eq. (S11), along with Eq. (S12), into the pressure jump Eq. (S15), and remove all the base state components. The Laplace pressure Eq. due to the perturbation then transforms into the following dimensionless

dispersion-relation with the dimensionless growth rate, $\bar{\sigma} = \frac{\sigma r_0}{U_0}$, and the dimensionless wavenumber, $\bar{k} = k$:

$$\begin{aligned}
&\frac{12\bar{\sigma}h_0}{\gamma}\left(\kappa_1\sqrt{n_1}\left(\frac{U_0}{h_0}\right)^{n_1} + \kappa_2\sqrt{n_2}\left(\frac{U_0}{h_0}\right)^{n_2}\right) = -\frac{12U_0}{\gamma}\left(\sqrt{n_1}\mu_1|_{r=r_0} + \sqrt{n_2}\mu_2|_{r=r_0}\right)\\
&-\frac{12\alpha r_0}{\gamma}\left(2\sqrt{n_1}\tau_{c1} + 2\sqrt{n_2}\tau_{c2} + \sqrt{n_1}\kappa_1\left(\frac{U_0}{h_0}\right)^{n_1} + \sqrt{n_2}\kappa_2\left(\frac{U_0}{h_0}\right)^{n_2}\right)\\
&+\bar{k}\left(\frac{12U_0}{\gamma}\left(\mu_2|_{r=r_0} - \mu_1|_{r=r_0}\right) + 2\alpha\cos\theta_c + \frac{{h_0}^2}{{r_0}^2}\right) - \frac{{h_0}^2}{{r_0}^2}\bar{k}^3,
\end{aligned} \tag{S16}$$

with the complex fluids' viscosity at the interface given as $\mu_j|_{r=r_0} = \left(\tau_{cj} + \kappa_j\left(\frac{U_0}{h_0}\right)^{n_j}\right)\frac{h_0}{U_0}$.

Consequently, with $\tau_{cj} = 0$, $n_j = 1$, $\kappa_j = \mu_j$ for simple Newtonian fluids, and defining $\lambda = \frac{\mu_1}{\mu_2}$ and $Ca = \frac{12U_0\mu_2}{\gamma}$, the dispersion relation transforms into:

$$\frac{\sigma r_0 Ca}{U_0}(1+\lambda) = -Ca\,(1+\lambda)\left(1 + \frac{\alpha r_0}{h_0}\right) + \bar{k}\left(2\alpha\cos\theta_c + \frac{{h_0}^2}{{r_0}^2} + Ca\,(1-\lambda)\right) - \frac{{h_0}^2}{{r_0}^2}\bar{k}^3, \tag{S17}$$

which is the same relation found by Al-Housseiny and Stone for simple Newtonian fluids [12].

To find the wavenumber at the maximum growth rate, we take the derivative of above Eq. (S16) w.r.t. $\bar{k}$ by setting $\frac{\partial\bar{\sigma}}{\partial\bar{k}} = 0$, we find the wavenumber of maximum growth ($\bar{k}_{max}$) equal to:

$$\bar{k}_{max} = \left(\frac{\frac{{h_0}^2}{{r_0}^2} + 2\alpha\cos\theta_c + \frac{12U_0}{\gamma}\left(\mu_2|_{r=r_0} - \mu_1|_{r=r_0}\right)}{3\frac{{h_0}^2}{{r_0}^2}}\right)^{\frac{1}{2}}. \tag{S18}$$

The resultant wavelength of maximum growth rate, $\lambda_{max} = 2\pi r/\bar{k}_{max}$ hence is:

$$\lambda_{max} = 2\pi r\left(\frac{3\frac{{h_0}^2}{{r_0}^2}}{\frac{{h_0}^2}{{r_0}^2} + 2\alpha\cos\theta_c + \frac{12U_0}{\gamma}\left(\mu_2|_{r=r_0} - \mu_1|_{r=r_0}\right)}\right)^{\frac{1}{2}}. \tag{S19}$$

Alternatively, from the dispersion-relation Eq. (S16), the interface would always be stable for a non-positive growth rate ($\bar{\sigma} \leq 0$) when the following stability criterion is fulfilled:

$$\begin{aligned}
&\Bigg[-\frac{12U_0}{\gamma}\left(\sqrt{n_1}\mu_1|_{r=r_0} + \sqrt{n_2}\mu_2|_{r=r_0}\right) - \frac{12\alpha r_0}{\gamma}\left(2\sqrt{n_1}\tau_{c1} + 2\sqrt{n_2}\tau_{c2} + \sqrt{n_1}\kappa_1\left(\frac{U_0}{h_0}\right)^{n_1} + \sqrt{n_2}\kappa_2\left(\frac{U_0}{h_0}\right)^{n_2}\right)\\
&+\bar{k}\left(\frac{12U_0}{\gamma}\left(\mu_2|_{r=r_0} - \mu_1|_{r=r_0}\right) + 2\alpha\cos\theta_c + \frac{{h_0}^2}{{r_0}^2}\right) - \frac{{h_0}^2}{{r_0}^2}\bar{k}^3\Bigg]\frac{\gamma}{12h_0\left(\kappa_1\sqrt{n_1}\left(\frac{U_0}{h_0}\right)^{n_1} + \kappa_2\sqrt{n_2}\left(\frac{U_0}{h_0}\right)^{n_2}\right)} \leq 0.
\end{aligned} \tag{S20}$$

[1] D. Bonn, H. Kellay, M. Brunlich, M. Ben Amar, and J. Meunier, Physica A **220**, 60 (1995).

[2] E. Corvera Poiré and M. Ben Amar, Phys. Rev. Lett. **81**, 2048 (1998).

[3] M. B. Amar and E. Corvera Poiré, Phys. Fluids **11**, 1757 (1999).

[4] A. Lindner, D. Bonn, and J. Meunier, Phys. Fluids **12**, 256 (2000).

[5] L. Kondic, P. Palffy-Muhoray, and M. J. Shelley, Phys. Rev. E **54**, R4536 (1996).

[6] Eur. Phys. J E Soft Matter **36**, 139 (2013).

[7] L. Kondic, M. J. Shelley, and P. Palffy-Muhoray, Phys. Rev. Lett. **80**, 1433 (1998).

[8] T. Chevalier, S. Rodts, X. Chateau, C. Chevalier, and P. Coussot, Phys. Rev. E **89** (2014).

[9] N. Maleki-Jirsaraei, A. Lindner, S. Rouhani, and D. Bonn, J. Phys.: Condens. Matter **17**, 1219 (2005).

[10] P. Coussot, J. Fluid Mech. **380**, 363 (1999).

[11] W. H. Herschel and R. Bulkley, Kolloid Z. **39**, 291 (1926).

[12] T. T. Al-Housseiny and H. A. Stone, Phys. Fluids **25**, 092102 (2013).
